# Cholera outbreak trends in Nigeria: policy recommendations and innovative approaches to prevention and treatment

**DOI:** 10.3389/fpubh.2024.1464361

**Published:** 2024-09-06

**Authors:** Stanley Eneh, Francisca Onukansi, Collins Anokwuru, Ogechi Ikhuoria, Gabriel Edeh, Sochima Obiekwe, Zakariya'u Dauda, Awoyemi Praise-God, Chizaramekpere Okpara

**Affiliations:** ^1^Community Health Department, Obafemi Awolowo University, Osun, Ile-Ife, Nigeria; ^2^Department of Public Health, Federal University of Technology, Owerri, Imo, Nigeria; ^3^Department of Biochemistry, University of Port Harcourt, Port Harcourt, Nigeria; ^4^Department of Medicine, University of Nigeria Teaching Hospital, Ituku-Ozalla, Enugu, Nigeria; ^5^Department of Medical Rehabilitation, Nnamdi Azikiwe University, Awka, Anambra, Nigeria; ^6^School of Medical Laboratory Science, Usmanu Danfodiyo University, Sokoto, Nigeria; ^7^Faculty of Veterinary Medicine, University of Ibadan, Ibadan, Nigeria

**Keywords:** cholera outbreak, Nigeria, innovative approaches, prevention, policy, cholera control

## Abstract

Cholera, an acute diarrheal infection from ingesting contaminated food or water, remains a significant public health threat in Nigeria, especially in areas lacking safe water and sanitation. Characterized by severe watery diarrhea, cholera can cause dehydration and death if untreated. Historical data shows cholera's endemic nature in Nigeria, with notable outbreaks since 1970, including major ones in 1991, 1999, 2010, 2018, and 2024. According to a descriptive study in Nigeria, the 1991 outbreak reported 59,478 cases and 7,654 deaths, with a Case Fatality Ratio (CFR) of 12.9%. In 2010, there were 41,787 cases and 1,716 deaths, with a CFR of 4.1% across 18 states, mainly affecting impoverished communities and children. The 2018 outbreak had 43,996 cases and 836 deaths, with a CFR of 2% in 20 states, a 240% increase from 2017. By mid-2024, there were 1,579 suspected cases and 54 deaths (CFR 3.4%) in 32 states. This paper evaluates cholera trends in Nigeria and proposes effective preventive and treatment strategies. Policy recommendations highlight the need for improved WASH infrastructure, enhanced surveillance, and rapid response mechanisms. Innovative approaches like case-area targeted interventions (CATI) and increased public health education are crucial for mitigating future outbreaks and achieving the goal of reducing cholera deaths by 90% by 2030.

## 1 Introduction

Despite the established knowledge of transmission pathways and preventative measures over the past 150 years, cholera continues to pose a significant public health challenge in many regions, particularly in Africa, Asia, and Central and South America (1). In the early twenty-first century, cholera continues to be a global concern, leading to large outbreaks in countries such as Haiti, Yemen, Nigeria, the Democratic Republic of Congo, the Dominican Republic, Egypt, Somalia, Bangladesh, Pakistan, the Philippines, China, Ghana, and Cameroon, as well as remaining endemic in numerous other nations (2). Approximately 2.9 million cholera cases occur annually, leading to around 95,000 fatalities globally, with most of these cases and deaths concentrated in low- and middle-income countries (3).

Notably, cholera serves as an indicator of inequality, disproportionately impacting the world's most impoverished populations. It predominantly affects individuals already rendered vulnerable by conditions of poverty and conflict (2). In 2021, cholera outbreaks were reported in 23 countries, predominantly in the WHO regions of Africa and the Eastern Mediterranean. This trend persisted into 2022, with cholera cases or outbreaks emerging in 30 countries spanning five of the six WHO regions (4). Nigeria, one of the countries in the WHO African region, continues to face persistent annual incidences of cholera. As of July 21, 2024, Nigeria had recorded over 4,809 suspected cases, resulting in 156 deaths with a case fatality ratio (CFR) of 3.2% across 35 states in Nigeria (192 local government areas) (5). This increasing trend necessitates a proactive measure to tackle the country's rising cholera infection cases. Hence, this paper aims to evaluate the trends of cholera incidences in the country while proposing effective, innovative approaches to preventing and treating infection.

## 2 Trends and epidemiology of cholera outbreaks in Nigeria

Since 1970, Nigeria has been endemic to cholera, experiencing significant outbreaks over the years. Notably, in 1991, a major outbreak resulted in 59,478 cases and 7,654 deaths, with a case-fatality ratio (CFR) of 12.9% (6). Another significant outbreak occurred in March 1999 in Kano State, subsequently spreading to Adamawa and Edo states by May, resulting in 26,358 cases and 2,085 deaths (6). Findings revealed that the 2010 cholera outbreak in Nigeria resulted in 41,787 cases and 1,716 deaths with a case-fatality ratio (CFR) of 4.1% across 18 states, highlighting the vulnerability of poor communities, particularly children, to the infection (4). In 2014, there were 4,996 reported cases of cholera, with a CFR of 2% (7). In 2018, epidemiological surveillance reported 43,996 cases and 836 deaths across 20 states between January 1st and November 19th, with a CFR of 2% (8), representing a 240% increase in cases compared to 2017. As of July 21, 2024, Nigeria had recorded over 4,809 suspected cases, resulting in 156 deaths with a case fatality ratio (CFR), of 3.2% across 35 states in Nigeria (192 local government areas) (5) (see Table 1). Of the suspected cases since the beginning of the year, the majority have occurred in children under the age of 5, followed by those in the 5–14 age group. Moreover, of all suspected cases, 50% are males and 50% are females (9). These studies have shown varying trends in the cholera case fatality ratio, dropping from 12.9% in the 1990's to 3.5% in 2024. However, this ratio is subject to change as the infection continues to spread across the country. Over the years, improved sanitation measures have contributed to the improvement in the case fatality ratio, but the ratios remain concerning and require urgent measures.

**Table 1:** Geographic distribution of cholera and affected regions in Nigeria [adopted from Nigeria Centre for Disease Control and Prevention (8, 24)].

**Year**	**States and local government area (LGA) affected respectively**	**Top 10 states affected**	**Number of cases**	**Number of suspected deaths**	**Case fatality ratio**
January to June 2024	32 states and 115 LGA	• Lagos • Bayelsa • Abia • Zamfara • Bauchi • Cross River • Ebonyi • Rivers • Delta	1,579	54	3.4%
2023	31 states and 166 LGA	• Zamfara • Crossriver • Katsina • Bayelsa • Ogun • Ebonyi • Niger • Jigawa • Adamawa	3,683	128	3.5%
2022	33 states and 271 LGA	• Borno • Yobe • Katsina • Gombe • Taraba • Kano • Cross River Bauchi • Zamfara • Jigawa	23,763	592	2.5%
2021	34 states and 435LGA	• Bauchi • Jigawa • Kano • Zamfara • Katsina • Sokoto • Kebbi • Borno • Yobe • Niger	111,062	3,604	3.2%
2019	7 states and 33LGA	• Adamawa Bayelsa • Kano • Katsina • Plateau • Ebonyi • Delta	1,583	22	1.38%
2018	20 states and 206 LGA	• Adamawa • Anambra • Bauchi • Borno • Ebonyi • FCT • Gombe • Jigawa • Kaduna • Kano • Katsina • Kebbi • Kogi • Kwara • Nasarawa • Niger • Plateau • Yobe • Sokoto • Zamfara	42,466	830	1.95%

## 3 Determinants and risk factors of cholera outbreaks in Nigeria

Socioeconomic factors like poverty and lack of education contribute significantly to cholera outbreaks in Nigeria. Cholera flourishes in regions where there is restricted availability of uncontaminated water and unsanitary living conditions that usually coincide with poverty. Cholera cases in Nigeria have been increasing significantly since the 1970's, primarily due to widespread poverty. Due to the lack of basic contemporary infrastructure, most of the populations residing in rural areas in Nigeria are very susceptible to disease outbreaks (8).

Regardless of the availability of basic infrastructure, cholera can still spread efficiently if people lack adequate personal and home hygiene practices. This underscores the importance of public education in understanding the causes and prevention of cholera, as it plays a crucial role in minimizing its transmission (10). The environmental factors contributing to cholera outbreaks are multifaceted and interconnected, primarily involving poor sanitation, a lack of potable water, and inadequate waste management. Studies have identified inadequate WASH (water, sanitation, and hygiene) services as a significant factor in cholera outbreaks in Nigeria (11, 12). A study done in Kano and Plateau States of Nigeria on the genetic relatedness of vibrio cholera found that contaminated wastewater used for irrigating vegetables contributes to cholera outbreaks (13). Charnley et al. highlighted the role of social and environmental extremes such as conflicts and climate events and established a strong association between conflict and increased cholera risk (14, 15).

Furthermore, climate change significantly contributes to cholera outbreaks in Nigeria by influencing environmental conditions that promote the spread of the disease. A study in the Funtua Local Government Area of Katsina State demonstrated a strong correlation between increasing temperatures and rainfall, and the prevalence of cholera in Nigeria from 1985 to 2014 was found to peak, particularly in August (16). Perez-Saez et al provide a comprehensive analysis of cholera seasonality in Sub-Saharan Africa, demonstrating that cholera usually peaks during the rainy season, which correlates with increased precipitation and flooding (17). Other studies in Kano and Ebonyi States of Nigeria noted distinct seasonal variations, with more cases occurring during the rainy season (18, 19).

## 4 Efforts in mitigating cholera outbreaks in Nigeria

Worldwide, cholera remains a persistent issue (20). In Sub-Saharan Africa (particularly Nigeria) and other developing nations, around 1.7 billion people still get their water from feces-polluted sources, leading to a significant number of deaths from cholera (21). Nigeria is currently experiencing a concerning and frightening increase in cholera cases, particularly among the most vulnerable (9, 12). This incidence is caused by several reasons, including but not limited to unsanitary living conditions and a lack of access to WASH services (water, sanitation, and hygiene) (22).

In 2017, the Global Task Force on Cholera Control (GTFCC) released A Global Roadmap to 2030 to reduce cholera-related deaths by 90% (21). Implementing this multi-sectoral public health strategy is necessary, as demonstrated by the 2018 outbreak, which resulted in 43,996 cases and 836 deaths (6). To address this, Nigeria has implemented various strategies, including public policy, surveillance, water purification, and hygiene (23). The country has established a National Cholera Multi-Sectoral Technical Working Group (TWG) to lead efforts in monitoring cholera incidence across all states and providing support to those affected. Additionally, ongoing surveillance is conducted nationwide through the routine Integrated Disease Surveillance and Response (IDSR) system and Event-Based Surveillance (EBS), ensuring the continuous tracking of cholera cases (24). In a recent time, through the combined efforts of Gavi, the World Health Organization, partners, the Nigeria Center for Disease Control, and the Borno State Ministry of Health, free cholera vaccines were provided to the affected areas in Nigeria to help with cholera prevention and preparedness (25).

However, a lack of knowledge about the basic causes of cholera in Nigeria has impeded prevention and control initiatives in some Nigerian communities (18). To maximize these efforts, a coordinated and unified educational package and approach to surveillance and response are necessary due to the endemic nature of cholera in Nigeria, which is exacerbated by socioeconomic and environmental factors (22).

## 5 Policy recommendations for effective cholera prevention and control

Nigerian cholera prevention and control initiatives will take a multi-pronged strategy. Previous study conducted in the country has emphasized the importance of cholera prevention methods among locals and the necessity of ongoing awareness initiatives (26). Nigerians are yet to meet the GTFCC cholera eradication policy and strategies. Although efforts are being made to meet the targets of the Global Task Force on Cholera Control (GTFCC) policy and target, there is a need to formulate an effective and innovative cholera reporting system policy in the country. These efforts would involve bolstering the reporting, response, and coordination axes. To stop the spread of cholera, case-area targeted interventions (CATI) are being used more frequently. They include suggestions for lowering the CATI radius, offering clear direction, securing manpower and supplies, enhancing community engagement, and improving surveillance (14). It is also important to emphasize the significance of safe drinking water and improving sanitary conditions (WASH) and also leverage the following policy recommendation to support already existing cholera control and prevention across the nation:

### 5.1 WASH policy

WASH has been an effective strategy used to control cholera globally. In 2018, the Nigerian government declared a state of emergency in the Water, Sanitation, and Hygiene (WASH) sector (27). This declaration served as a call to action. The Nigerian government reinforced its commitment to improving access to WASH services by launching the National Action Plan (NAP), a 13-year strategy for the revitalization of the Nigerian WASH sector. Unfortunately, there is no active WASH monitoring policy for sustainable and safely managed WASH services across Nigeria, in alignment with the Sustainable Development Goal 6, which emphasizes the crucial need for ensuring “***clean water and sanitation for all***.” As of March 2024, Lagos State, one of the 36 states in Nigeria, had launched a WASH policy to regulate and support SDG 6 (28). There is a need to establish WASH policies nationwide to provide clean drinking water, which would directly and indirectly serve as a basic step for the control of cholera and other related diseases in Nigeria.

### 5.2 National cholera plan and policy development

To the best of our knowledge, Nigeria has yet to establish a comprehensive national cholera policy and plan. Reports indicate that Nigeria experienced a cholera outbreak as early as 1972. In late 2010, a severe outbreak began in northern Nigeria, resulting in over 2,800 cases and more than 780 deaths, with subsequent outbreaks occurring in various parts of the country (6, 29, 30). Despite significant efforts to eliminate and control these outbreaks, the recurring nature of cholera in Nigeria underscores the urgent need for a national cholera policy and elimination plan.

However, according to the Global Task Force on Cholera Control (GTFCC), cholera transmission can be halted and cholera deaths significantly reduced through the implementation of coordinated multi-sectoral interventions in line with a national cholera plan (31). It was reported that Nigeria had a draft cholera work plan for 2022, and the GTFCC encouraged the establishment of a comprehensive national cholera plan during a national cholera preparedness, prevention, and response training in February 2022 (32, 33). Given the past and ongoing cholera outbreaks in Nigeria, more intensive interventions are necessary for a national cholera elimination plan. These should include community engagement, enhanced Water, Sanitation, and Hygiene (WaSH) practices, the use of Oral Cholera Vaccines (OCV), and the strengthening of health systems. Therefore, we recommend the development of a national cholera plan and policy in Nigeria, guided by the 2020 GTFCC cholera plan (31, 34).

## 6 Innovative approaches to cholera prevention and treatment

Enhancing cholera preparedness and control in Nigeria would require inter-sectoral collaborations and initiatives.

### 6.1 Technological advancement

Notably, there have been significant technological advancements in water purification and sanitation. Nanotechnology, ozone therapy, electrocoagulation, and contemporary traditional water treatment techniques have all been integrated into Nigeria's water purification and sanitation developments. Using materials such as carbon nanotubes, alumina fibers, zinc oxide nanoparticles, and nano titanium oxide, nanotechnology provides effective water filtration (35). Ozone treatment, produced by corona discharge or water electrolysis, can effectively remove odor-producing chemicals and disinfect water (36). Electrocoagulation has been developed to sterilize wastewater and remove impurities such as oil-water emulsions and heavy metals (37). Despite these technological advancements, Nigeria's modern traditional water treatment plants face challenges with maintenance, power supply, and engaging trained workers. These issues highlight the necessity for effective management and automation to support the fight against cholera outbreaks in Nigeria (38).

### 6.2 Establish active reporting system

Mobile health and telemedicine are other technological advancements that have demonstrated potential for improving water sanitation and cholera control. Mobile telemedicine initiatives have proven to be successful in facilitating virtual connections between health practitioners and underserved communities. One such initiative, the “one2one” mobile telemedicine app, is connected through Nigeria's communication satellite system, and it has improved access to medical specialists in remote areas (39). Furthermore, community health workers in rural Nigeria are now able to diagnose and treat diseases through the use of mHealth software on cell phones. Additionally, identifying social innovations through crowdsourcing issues could lead to the development of mobile clinics and digital health solutions (40). Nigeria could enhance its cholera reporting system by leveraging the mHealth and telemedicine platforms.

### 6.3 Community-driven initiatives and local innovations

The response to cholera outbreaks in Nigeria should include community-based initiatives and local innovations. Studies conducted in three states in Nigeria have emphasized the benefits of community-driven initiatives in disease control (26, 29, 41). By prioritizing safe water, sanitation, and hygiene practices, incorporating health lectures and awareness campaigns, and promoting cholera prevention techniques among residents, cholera control becomes achievable (25). Additionally, measures like active case finding, case management, and ensuring water cleanliness should be implemented to control epidemics and reduce morbidity and mortality rates (3). To effectively combat cholera epidemics and develop sustainable prevention strategies, Nigeria should enhance education efforts, foster community engagement, and ensure access to clean water.

### 6.4 Strategic public campaigns and education programs

Public campaigns and education programs are recognized public health strategies for eradicating diseases and controlling outbreaks globally (42). Health awareness marketing educates individuals and communities about potential health risks and symptoms, promoting the early detection and prevention of serious health conditions (43, 44). Given the current cholera outbreak in Nigeria, there is an urgent need for strategic action plans through public campaigns and education programs. The Nigerian health authorities should establish a “***Cholera Day***” to serve as a reminder of the national effort to end cholera and to strengthen the national campaign and awareness efforts.

### 6.5 Strengthening community-based surveillance

Individuals living in remote areas are particularly susceptible to cholera due to poor awareness and weak Integrated Disease Surveillance and Response (IDSR) systems within their communities. Although Nigeria adopted IDSR as its public health surveillance strategy in 2001, its effectiveness varies significantly across different states (3).

**Figure 1:**
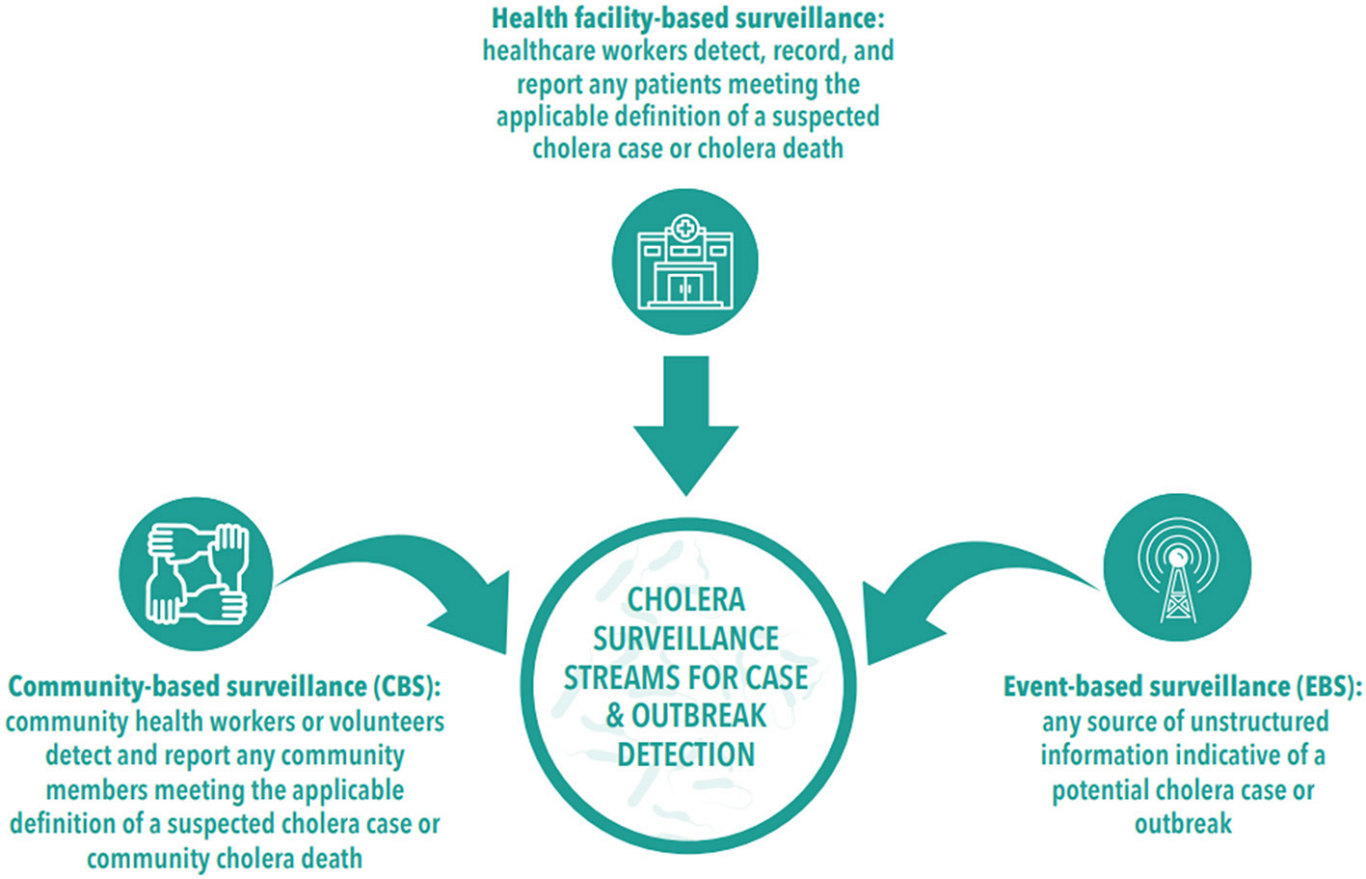
Cholera surveillance stream [adopted from Global Task Force on Cholera Control (34)].

The poor functionality of this surveillance system has hindered effective outbreak responses in Nigeria (45, 46). This gap in active community-based surveillance, directly and indirectly, threatens the achievement of the three strategic axes of the global cholera roadmap, which aims to end cholera worldwide by 2030 (47). The Nigerian health authorities must implement the Global Cholera Roadmap, as recommended by the Global Task Force on Cholera Control (GTFCC), to reduce cholera deaths by 90% in the country (47). This requires strengthening both national and local health surveillance systems and enhancing cholera surveillance streams, which include health facility-based, community-based, and event-based surveillance (34). Health facility-based and community-based surveillance should routinely detect suspected cholera cases, while event-based surveillance should identify cholera signals (see Figure 1).

### 6.6 Enhancing water, sanitation, and hygiene for cholera control

Water, Sanitation, and Hygiene (WaSH) have been crucial in combating cholera outbreaks in Nigeria since 2017, aligning with UNICEF's 2018 strategy recommendations for cholera control (48). The Nigerian government has effectively employed WaSH as its primary strategy for cholera control, receiving substantial backing from the Global Task Force on Cholera Control (GTFCC) (32, 49). This approach has proven effective in various parts of the world for cholera control (50, 51). We advocate for a sustainable, integrated WaSH plan that includes developing a comprehensive water supply strategy and adopting the Integrated Behavioral Model for Water, Sanitation, and Hygiene (IBM-WaSH), alongside other WaSH models and frameworks. Such initiatives will significantly bolster the efficacy of cholera control policies, enhance overall health outcomes, and fortify national health security.

## 7 Limitation of study

This study primarily utilizes historical data and reports from various sources, which may present inconsistencies in evidence-based findings. The limited access to the cholera data reporting system in Nigeria further challenges the adequacy of the data reported in this study. Therefore, future research should consider employing primary, secondary data analysis, as well as systematic or scoping reviews to accurately assess trends in cholera outbreaks in Nigeria. This approach would further support evidence-based practices in cholera outbreak control and management.

## 8 Conclusion

Cholera remains a significant public health threat in Nigeria, exacerbated by inadequate water and sanitation infrastructure, poor community-based surveillance, and socio-economic disparities. The recurring challenges of cholera outbreaks highlight the urgent need for a comprehensive and coordinated approach. This approach should combine strengthened community-based surveillance systems, enhanced WASH practices, and robust community engagement, all of which are critical for controlling and preventing future outbreaks.

Implementing the Global Cholera Roadmap recommended by the Global Task Force on Cholera Control (GTFCC) is also crucial to reducing cholera deaths by 90% in Nigeria by 2030. Nigerian health authorities must adopt sustainable and integrated public health interventions that prioritize vulnerable populations, ensuring equitable access to safe water, improved sanitation, and effective disease surveillance. The adoption of behavioral models for hygiene practices will significantly bolster cholera control policies, improve health outcomes, and fortify national health security. Moreover, collaborative efforts between government agencies, international organizations, and local communities are essential to achieving a cholera-free Nigeria.

## Data Availability

The original contributions presented in the study are included in the article/supplementary material, further inquiries can be directed to the corresponding author.
